# Tween-80 improves single/coaxial electrospinning of three-layered bioartificial blood vessel

**DOI:** 10.1007/s10856-022-06707-x

**Published:** 2022-12-31

**Authors:** Chuang Wu, Haixiang Wang, Jin Cao

**Affiliations:** 1 College of Mechanical Engineering, Yangzhou University, No. 196 West Huayang Road, Yangzhou, 225127 China; 2 Nantong Fuleda Vehicle Accessory Component Co., Ltd, Nantong, 226300 China

## Abstract

Electrospinning is a promising technique for preparing bioartificial blood vessels. Nanofibers prepared by electrospinning can simulate the structure of extracellular matrix to promote cell adhesion and proliferation. However, thorn-like protrusions can appear as defects on electrospun scaffolds and coaxial electrospun nanofibers often have no clear core/shell structure, which can seriously affect the quality of bioartificial blood vessels. To address these problems, Tween 80 is added to the electrospinning solution, which results in a stable Taylor cone, eliminates the thorn-like protrusions on electrospun bioartificial blood vessels, and reduces interfacial effects due to different core/shell solutions during coaxial electrospinning. Simulations, biomechanical tests, and in vivo studies were performed. The results demonstrate the excellent mechanical properties and biocompatibility of the bioartificial blood vessel. This research provides a useful reference for optimizing the electrospinning process for fabricating bioartificial blood vessels.

**Graphical Abstract:**

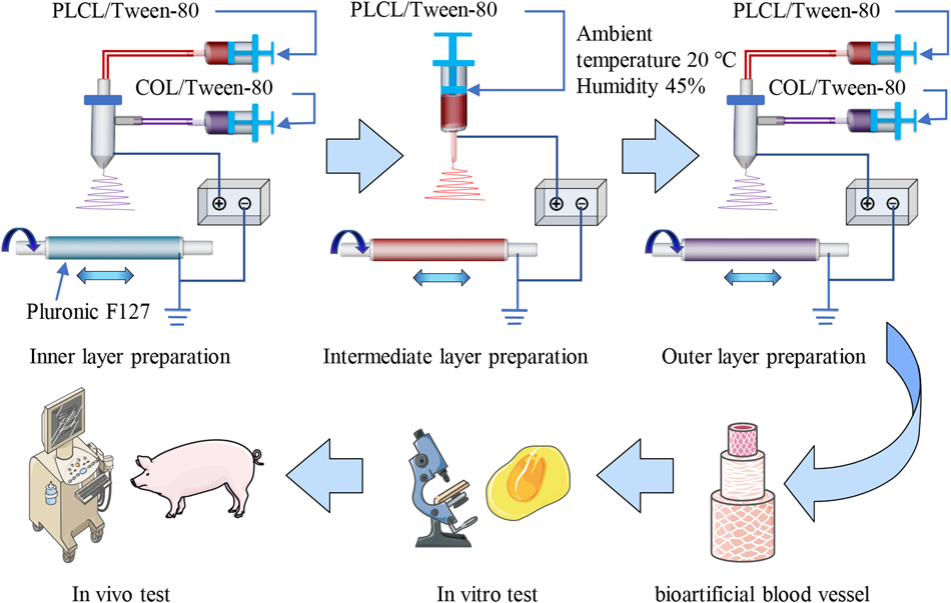

## Introduction

Vascular diseases caused by malignant tumors, infections, trauma, congenital malformations, genetics, etc., seriously threaten human health. The World Health Organization (WHO) estimates that by 2030, each year, 23.6 million people will die of cardiovascular disease, which is rapidly becoming a leading cause of mortality worldwide [1, 2]. Blood vessel transplantation is often used to treat patients with well-tolerated cardiovascular diseases [3, 4]. Although blood vessel replacements are long-lasting and reliable, autologous blood vessels can cause secondary damage to the body, while blood vessel replacements from allogeneic sources carry the risk of immune rejection. Currently, bioartificial blood vessels are the most widely used replacement [5–7].

Nanofibers prepared by electrospinning have a high specific surface area, and the fiber diameter is smaller than the diameter of a human cell. Nanofiber scaffolds can simulate the outer matrix structure of cells, and are conducive to cell adhesion and proliferation. Thus, electrospinning presents a relatively promising technique for fabricating bioartificial blood vessels [8, 9]. Collagen (COL) is a structural protein of the extracellular matrix that can be absorbed and degraded by the body, however, its mechanical properties are poor [10, 11]. Poly-L-lactide-caprolactone (PLCL, 75/25), a copolymer of poly-L-lactide (PLLA) and polycaprolactone (PCL), can be absorbed and degraded by the human body and offers good biocompatibility along with the high strength of PLLA and flexibility of PCL [12, 13]. Tween 80, also known as polysorbate 80, is a non-ionic, electrically conductive surfactant material that can reduce surface tension and interfacial effects of organic solvents without destroying the structure of natural biological materials like collagen. In addition, Tween 80 is approved by The US Food and Drug Administration (FDA) for intravenous injection [14, 15]. Pluronic F127 (F127) is a temperature-sensitive material that is highly soluble in water and ethanol. The hydrophilic non-ionic surfactant is liquid between 0 and 4 °C and gelatinous at room temperature. Moreover, its monomers can be eliminated through the kidneys and will not accumulate in the body and it is approved by the FDA as a drug carrier [16, 17].

In this study, a novel electrospinning process was used to prepare bioartificial blood vessels. As the thickness of the electrospinning layer increases during the electrospinning process, charges accumulate on the surface that are not easily discharged, leading to thorn-like protrusions on the bioartificial blood vessel [18, 19]. With coaxial electrospinning, the use of different core and shell solutions can cause interfacial effects, resulting in coaxial nanofibers with no clear core/shell structure [20–22]. Coating the receiving shaft with F127 and adding Tween 80 to the electrospinning solution ensures the bioartificial blood vessel can be easily removed from the shaft and thorn-like protrusions are eliminated. In this study, nanofibers with a clear core/shell structure were obtained. Macro and microscopic morphological observations, simulations, and mechanical tests were carried out, and the excellent biomechanical properties of the bioartificial blood vessel were demonstrated. Cytocompatibility tests and in vivo experiments were performed and the results provide a useful reference for the preparation and optimization of bioartificial blood vessels.

## Materials and methods

### Materials

Poly(L-lactide-co-ε-caprolactone) (PLCL, 50:50, Mw = 3 × 105 Da), collagen (COL, Mw = 0.8–1 × 105 Da), Pluronic F127 (F127, Mw = 12,600 Da), polysorbate 80 (Tween 80, cell culture grade), 2,2,2-trifluoroethanol A (TFEA), N-(3-dimethylaminopropyl)-N’-ethylcarbodiimide (EDC, Mw = 191.70 Da), N-hydroxysuccinimide (NHS, Mw = 115.09 Da), dichloromethane (DCM), 1,1,1,3,3,3-hexamethyldisilazane (HMDS), and ethanol were purchased from Aladdin Biochemical Technology, Shanghai, China.

Human umbilical vein endothelial cells (HUVECs), cell culture medium (RPMI 1640), fetal bovine serum, 4-(2-hydroxyethyl)-1-piperazineëthanesulfonic acid (HEPES) buffer, L-glutamine, streptomycin, sodium-bicarbonate (NaHCO3), phosphate-buffered saline (PBS), penicillin, Live/Dead cell staining kit, and Cell Counting Kit-8 (CCK-8) were purchased from Zhongqiaoxinzhou Biotech, Shanghai, China.

### Methods

#### Preparation of bioartificial blood vessels

The air temperature was adjusted to 20 °C and the humidity to 45%, and the stainless-steel receiving shaft (length 200 mm, diameter 4.5 mm) was evenly coated with F127 hydrogel (35 wt%, with deionized water as the solvent). First, the inner layer of the bioartificial blood vessel was prepared by electrospinning using a PLCL solution (8 wt%, solvent is TFEA/DCM 1:1 v/v) as the core layer and COL solution (7.5 wt%, solvent is TFEA) as the outer shell of the nanofibers. The receiver shaft rotated at 80 rpm and a horizontal reciprocating speed of 1.5 mm/min was maintained to uniformly collect the electrospun fibers. The feeding speed of the core/shell solutions was 1 μL/min and the vertical distance from the nozzle to the receiving shaft was 12 cm. The voltage was 14 kV and the coaxial electrospinning time was 3 h. Next, the middle layer of the bioartificial blood vessel was prepared by uniaxial electrospinning at a voltage of 12 kV with a feeding speed of 0.35 μL/min and vertical distance between the nozzle and the receiving shaft of 12 cm. The uniaxial electrospinning time was 4 h and the rest of the conditions were the same as those used for electrospinning the inner layer, and the PLCL fibers were uniformly collected on the receiving shaft. Finally, the outer layer of the bioartificial blood vessel was prepared using the same process and preparation time as the inner layer.

After the electrospinning process was complete, the three-layer bioartificial blood vessel was placed, together with the receiving shaft, at 0–4 °C to liquefy the F127 to allow the bioartificial blood vessel to smoothly slide off of the receiving shaft. Residual F127 on the bioartificial blood vessel was removed with absolute ethanol, then the scaffold was cross-linked in EDC/NHS solution (1.5%, m/m = 5:2, with absolute ethanol as the solvent) for 48 h and vacuum dried for 72 h to obtain a bioartificial blood vessel without Tween-80. Then, 1 wt% Tween-80 was added to PLCL solution and COL solution and a second bioartificial blood vessel with Tween-80 was obtained using the same preparation conditions.

**Scheme 1:**
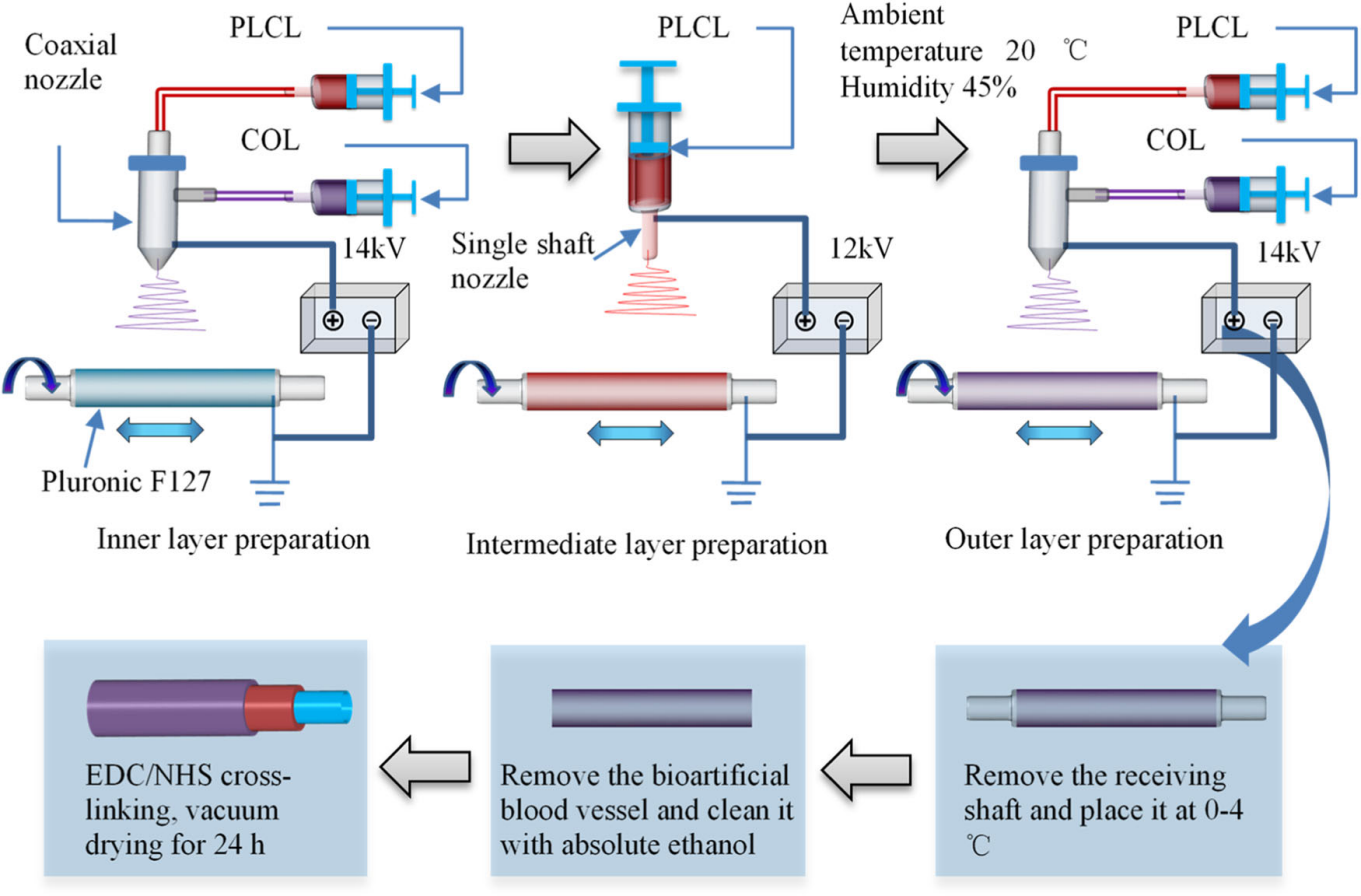
Schematic of compound forming process for fabricating bioartificial blood vessel

A schematic diagram of the composite forming process for bioartificial blood vessels is presented in Scheme 1.

#### Macro and micro morphological observations

The wall thickness and inner diameter of the bioartificial blood vessels were measured with vernier calipers. The morphology of the single/coaxial nanofibers was observed using a scanning electron microscope (SEM; SU-1510, Hitachi, Japan) and the core/shell structure was observed with a transmission electron microscope (TEM; JEM-2010F, JEOL, Japan). Fifty nanofibers were selected in each image and the diameter of each nanofiber was measured in ImageJ (v1.8.0, National institutes of health, Bethesda, MD, USA). The Taylor cone structure of the electrospinning process was observed with a high-speed camera (SpeedCam Mini-1, HSVISION, GER).

#### Modeling of bioartificial blood vessel

The bioartificial blood vessel was modeled using the following steps:

1. The inner diameter of the bioartificial blood vessel was assumed to be 4.5 mm, the length was assumed to be 30 mm, and the wall thickness was set as 1 mm. The height of thorn-like protrusions was 0.5 mm.
2. SolidWorks 2021 was used to model the bioartificial blood vessels using a standard mesh (Fig. 1).

**Fig. 1:**
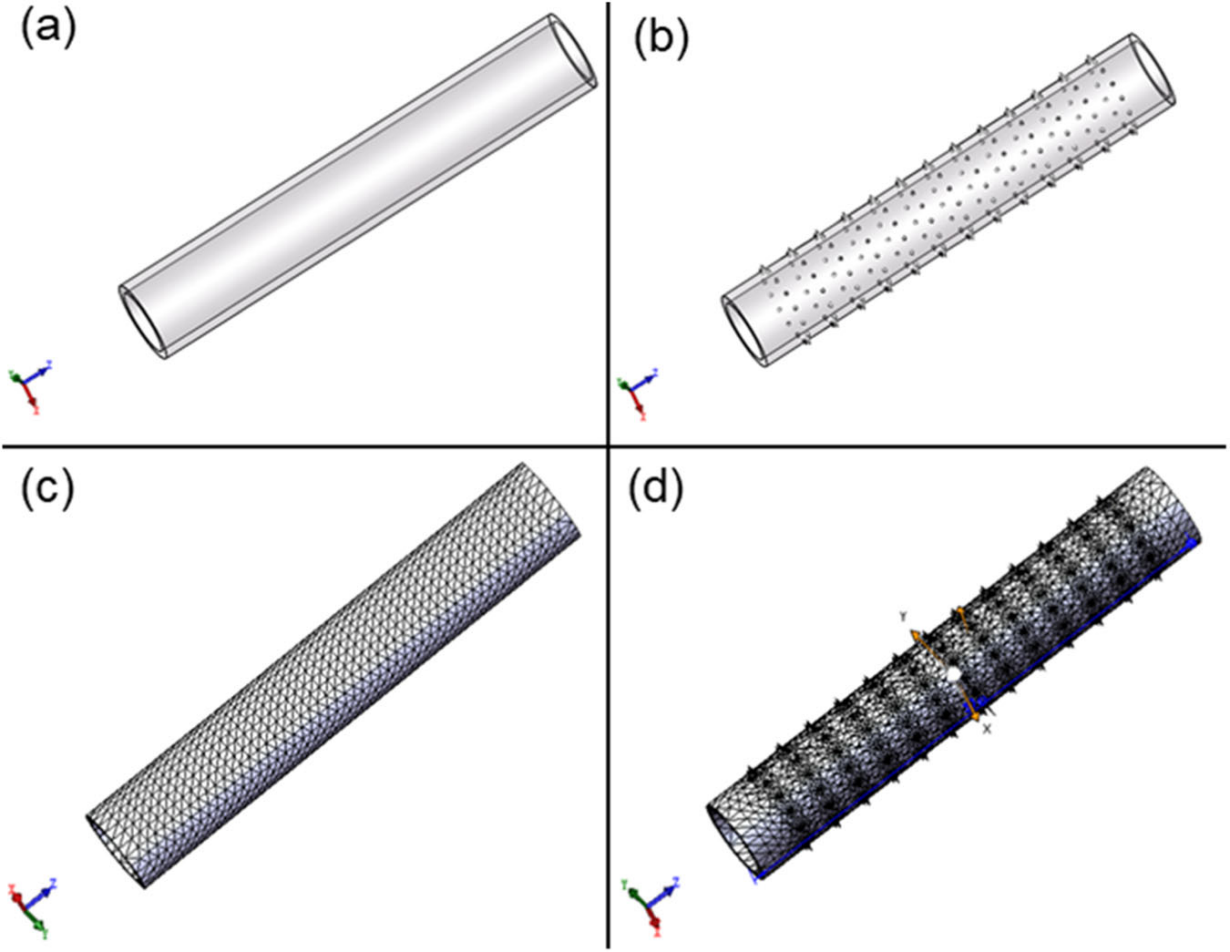
Modeling of bioartificial blood vessels without and with thorn-like protrusions: **a** Model of a bioartificial blood vessel without thorn-like protrusions. **b** Model of a bioartificial blood vessel with thorn-like protrusions. **c** Mesh of a bioartificial blood vessel without thorn-like protrusions. **d** Mesh of a bioartificial blood vessel with thorn-like protrusions
3. Inertial unloading and the direct sparse solver were used to simulate the axial forces acting on the bioartificial blood vessel. The axial force on both ends of the bioartificial blood vessel was 20 N. Inertial unloading and the FFEPlus solver were used to simulate the radial forces acting on the bioartificial blood vessel. The pressure on the inner surface was 1 MPa. The same mechanical loading method was adopted for bioartificial blood vessels with and without thorn-like protrusions.

#### Mechanical testing

1. Axial tension test
  A bioartificial blood vessel was cut to a length of 40 mm, soaked in deionized water for 10 min, then both ends were fixed on the chuck of a tensile testing machine (Songdun Machine Equipment, Shanghai, China) in an incubator at 37 °C. The bioartificial blood vessel was then stretched at a speed of 1/min in the axial direction until the bioartificial blood vessel was disconnected (Fig. 2a).

**Fig. 2:**
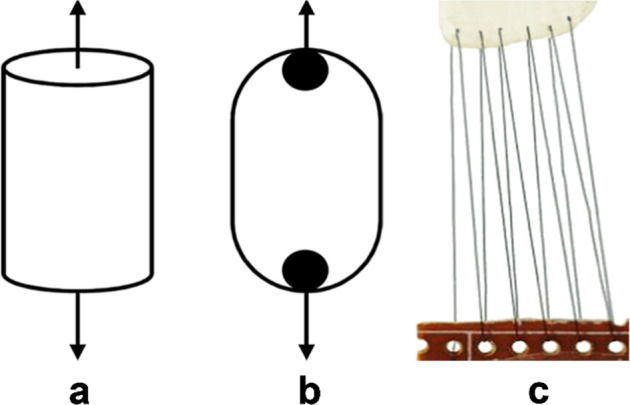
Schematic diagram of testing methods for assessing mechanical properties of the vascular scaffolds: **a** Axial stretching. **b** Radial stretching. **c** Suture strength testing
2. Radial tension test
  A bioartificial blood vessel was cut to a length of 10 mm, and soaked in deionized water for 10 min, then two U-shaped steel rods with a diameter of 2 mm were passed through the bioartificial blood vessel in an incubator at 37 °C. The steel rod was fixed to the clamp of the tensile testing machine. The bioartificial blood vessel was stretched at a speed of 2 mm/min until break (Fig. 2b).
3. Suture strength test
  A bioartificial blood vessel was soaked in deionized water for 10 min, then one end was sutured to the end of a plastic plate using a 6–0 medical polyester surgical thread in a constant temperature box at 37 °C. The other end of the bioartificial blood vessel was connected to the other end of the plastic plate and the plates were fixed to the chuck of the tensile testing machine. The bioartificial blood vessel was stretched at a speed of 2 mm/min until it disconnected (Fig. 2c).
4. Burst pressure test
  Using Laplace’s law [23, 24], the burst pressure of the bioartificial blood vessel can be calculated from the radial tensile strength. According to Laplace’s law, for a constant-diameter vessel the burst pressure is positively correlated with wall thickness (Equation 1):
  1 $$P_{burst} = \frac{{\tau \times \delta _C}}{\gamma }$$
  where P_burst_ is the burst strength (Pa), τ is the wall thickness of the bioartificial blood vessel (mm), δ_c_ is the radial limit stress (Pa), and γ is the radius of the bioartificial blood vessel (mm).
5. Swelling test

The swelling of the bioartificial blood vessels was tested by the weighing method. The bioartificial blood vessels were cut into sections with a length of 40 mm and divided into five groups. Each group was immersed in PBS for 1 h. Subsequently, the mass before and after drying was measured. The swelling rate was calculated as follows:

2 $${{{\mathrm{Swelling}}}}\;{{{\mathrm{Ratio}}}}\left( \% \right) = \left[ {\left( {{{{\mathrm{m}}}}1 - {{{\mathrm{m}}}}2} \right)/{{{\mathrm{m}}}}2} \right] \times 100\%$$

where m1 is the mass before drying (g) and m2 is the mass after drying (g).

#### Cytocompatibility test

1. Pre-treatment
  A bioartificial blood vessel was cut into multiple sections with lengths of 10 mm, which were then soaked in 75% alcohol for 2 h. The samples were removed from the alcohol in a sterile environment and rinsed in PBS, then rinsed again and soaked in cell culture medium and sterilized under ultraviolet radiation for 24 h.
2. Cell seeding
  For all experiments, HUVECs were used at the third generation. A concentration of 5.0 × 10^7^ cells/ml in 0.5 ml was seeded into the lumen of the bioartificial blood vessel, then the scaffold was placed in an incubator. The bioartificial blood vessel was rotated by 90° every 30 min. After 4 h, the cells had adhered to the wall and culture medium was added to the culture dish. The medium was changed every 2 days.
3. Cell staining
  The Live/Dead staining kit (Biovision, Inc., San Francisco, CA, USA) was used to stain cells on the bioartificial blood vessels [25–27]. Briefly, 1 μl of calcein-acetoxymethylester (calcein-AM) and 1 μl of propidium iodide (PI) were mixed with 1 ml of PBS. The solution was added to each scaffold and cells were incubated for 20 min at 37 °C. The fluorescently labelled cells were then observed under an inverted fluorescence microscope (Eclipse Ti-U, Nikon, Japan).
4. Cytotoxicity test
  The CCK-8 kit was used to assess toxicity by indirectly measuring the proliferation of cells on the bioartificial blood vessels [28–30]. Cells were seeded at a density of 10^4^ cells per well into 96-well plates in three experimental groups: scaffolds crosslinked with EDC/NHS (Group A), tissue culture plastic (Group B), and no scaffold and no cells (control; Group C). Briefly, 200 μl of cell suspension was added to each well and the HUVECs were cultured for 2, 4, or 7 days at 37 °C and 5% CO_2_. At each time point, the medium was removed, 10 μl of CCK-8 was added to each well, and the cells were incubated for a further 3 h. Light absorption values (OD values) were measured at 450 nm using a microplate reader (Infinite 200 Pro, Tecan, Switzerland).
5. Cell morphology

The cells were fixed in 2.5% glutaraldehyde, then soaked in ethanol (25%, 50%, 75%, 85%, 95%, and 100% for 5 min each) followed by hexamethyldisilazane (3:1, 1:1, 1:3, and 0:1 for 10 min each), then air-dried. The dehydrated samples were then sprayed with gold and the morphology of the cells on the bioartificial blood vessels was observed by SEM [31–33].

#### In vivo test

1. Experimental animals
  Male Shanghai white pigs weighing 60 kg were provided by Shanghai Junda Pet Clinic Co., Ltd., which is approved for the sanitation and quarantine of experimental pigs. All animal experiments were performed in accordance with ethical requirements. With the technical support of Shanghai Changhai Hospital, bioartificial blood vessels were implanted into the pigs to assess their biocompatibility.
2. Bioartificial blood vessel implantation
  1. The bioartificial blood vessels were soaked in 75% medical alcohol for 48 h, then cleaned in sterile saline to remove any residual alcohol. All surgical instruments were sterilized with high-temperature, high-pressure steam.
  2. The pigs were subjected to fasting for 12 h prior to the operation, injected with atropine (0.05 ml/kg), then intravenously injected with Shutai 50 (3 mg/kg) to anesthetize them. During the operation, an intravenous drip was used to provide sodium lactate ringer’s injection (200–300 ml) to maintain the acid-base balance of the body fluid.
  3. The anesthetized pigs were fixed on a special operating table in the supine position, and the tissue and muscle were cut with a scalpel to fully expose the femoral artery. After clamping the blood vessel with two arterial clamps, the middle blood vessel was cut off, a length of 40 mm was reserved, and end-to-end anastomosis was used to suture the bioartificial blood vessel to the remaining blood vessel. After the operation was complete, the arterial clip was released. If no leakage was observed, the incision was closed layer by layer.
  4. Color Doppler ultrasound (Philips Healthcare, Andover, MA, USA) was used to detect blood flow through the bioartificial blood vessel [34, 35].
  5. After the operation, the animals were monitored by Shanghai Junda Pet Clinic Co., Ltd.
3. Removal of bioartificial blood vessel
  1. During in vivo culture of the bioartificial blood vessels, the pigs were observed once a week.
  2. At the end of the experiment, the pigs were anesthetized and fixed on the operating table in the supine position to expose the surgical scar.
  3. Color Doppler ultrasound was performed on the surgical scar.
  4. The surgical site was sterilized with iodophor and extraneous tissue was peeled off of the wound using a scalpel. The two ends of the blood vessel were clamped with arterial clamps, then the bioartificial blood vessel was removed and any remaining blood on the surface was washed off with saline.
  5. The bioartificial blood vessel was cut along the center to observe any endothelial hyperplasia or thrombus.
  6. The extracted samples were embedded in standard wax, sectioned, and stained with hematoxylin-eosin (HE) and vascular endothelial growth factor (VEGF) [36, 37].

#### Statistical analysis

All data are presented as mean ± one standard deviation (SD) of n samples for each experimental group. Groups were compared using one-way analysis of variance (ANOVA) to determine significance [38, 39]. Differences between groups were considered significant when *p* < 0.05. Analyses were performed in SPSS (v26.0, SPSS, Inc., Chicago, USA).

## Results and discussion

### Bioartificial blood vessel appearance

**Fig. 3:**
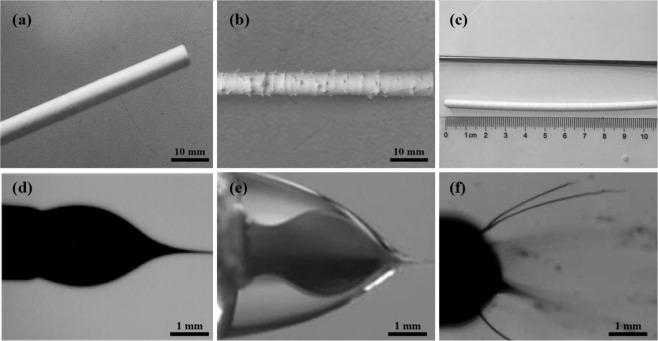
Macroscopic morphology of bioartificial blood vessels: **a** Without thorn-like protrusions. **b** With thorn-like protrusions. **c** Receiving shaft and bioartificial blood vessel. **d** Stable uniaxial Taylor cone structure. **e** Stable coaxial Taylor cone structure. **f** Unstable Taylor cone structure

When 1 wt% Tween 80 was added to the electrospinning solution, the surface of the bioartificial blood vessel exhibited no thorn-like protrusions (Fig. 3a), and it presented a stable single/coaxial Taylor cone structure. This was mainly due to that Tween 80 can reduce the surface tension of the electrospinning solution and improve the stability of the electrospinning process (Fig. 3d, e). Without Tween 80, objective thorn-like structures were observed (Fig. 3b) and the Taylor cone structure was unstable (Fig. 3f). By coating the surface of the receiving shaft with F127 hydrogel prior to electrospinning, the bioartificial blood vessel can be easily removed from the receiving shaft after liquefying the F127 at 0–4 °C. After removing the scaffold from the shaft, no wrinkles were observed on the surface (Fig. 3c).

**Fig. 4:**
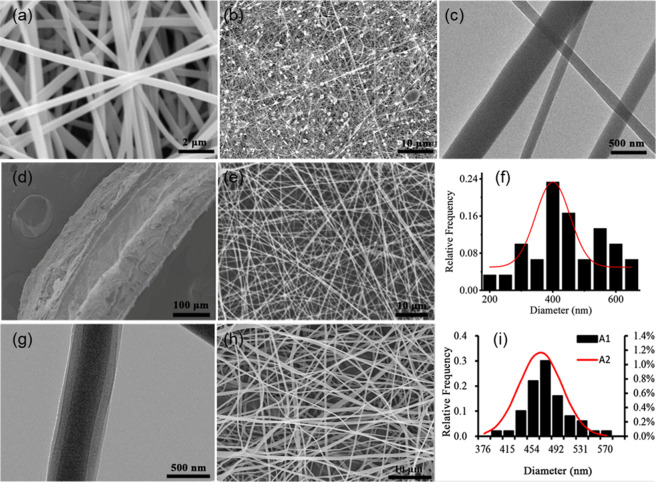
Microscopic morphology of the bioartificial blood vessels: **a** Uniform nanofiber filaments. **b** fibers with beaded structure. **c** Lack of core/shell structure. **d** Three-layer structure of bioartificial blood vessel. **e** Representative SEM image of uniaxial electrospun nanofiber. **f** Uniaxial electrospun fiber diameter distribution. **g** Representative TEM image of coaxial electrospun nanofiber. **h** Representative SEM image of coaxial electrospun nanofibers. **i** Coaxial electrospun fiber diameter distribution

The bioartificial blood vessel has a three-layer structure. The thickness of the outer layer is 196 ± 1.24 μm, the middle layer is 150 ± 1.31 μm, and the inner layer is 204 ± 1.72 μm (Fig. 4d). When 1 wt% Tween 80 is added to the electrospinning solution, the nanofiber filaments no longer have a beaded structure (Fig. 4a, e, h). The average diameter of uniaxial nanofibers is 400 ± 21 nm (*n* = 50) (Fig. 4f), and the average diameter of coaxial nanofiber filaments is 465.11 ± 34.04 nm (*n* = 50) (Fig. 4i). The diameters of the single/coaxial nanofibers follow a normal distribution. Coaxial nanofiber filaments were collected on a copper net and observed using TEM. A clear core/shell structure can be observed, and the diameter of the core layer is uniform (Fig. 4g), indicating that the coaxial electrospinning process has excellent stability. When Tween 80 was not added to the electrospinning solution, the nanofiber filaments exhibited a beaded structure (Fig. 4b), and the coaxial nanofibers did not have a clear core/shell structure (Fig. 4c).

From the above analysis, it can be concluded that adding Tween 80 to the electrospinning solution results in bioartificial blood vessels without thorn-like protrusions. Moreover, the nanofiber filaments were of uniform thickness with no beading and coaxial nanofibers with a clear core/shell structure were obtained.

### Simulation results

**Fig. 5:**
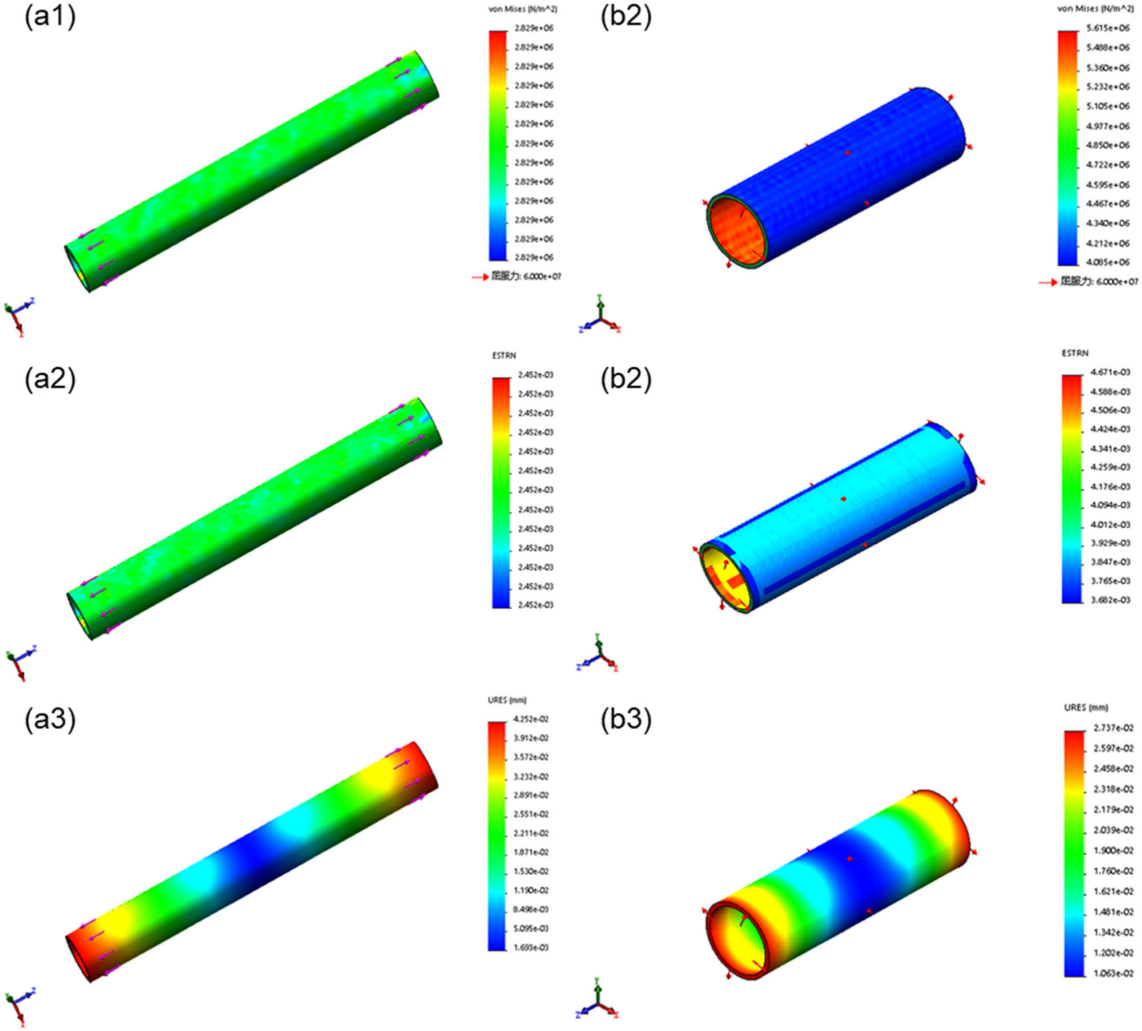
Mechanical simulation of bioartificial blood vessel without thorn-like protrusions: **a1** Axial stress, **a2** Axial strain. **a3** Axial displacement. **b1** Radial stress. **b2** Radial strain. **b3** Radial displacement

**Fig. 6:**
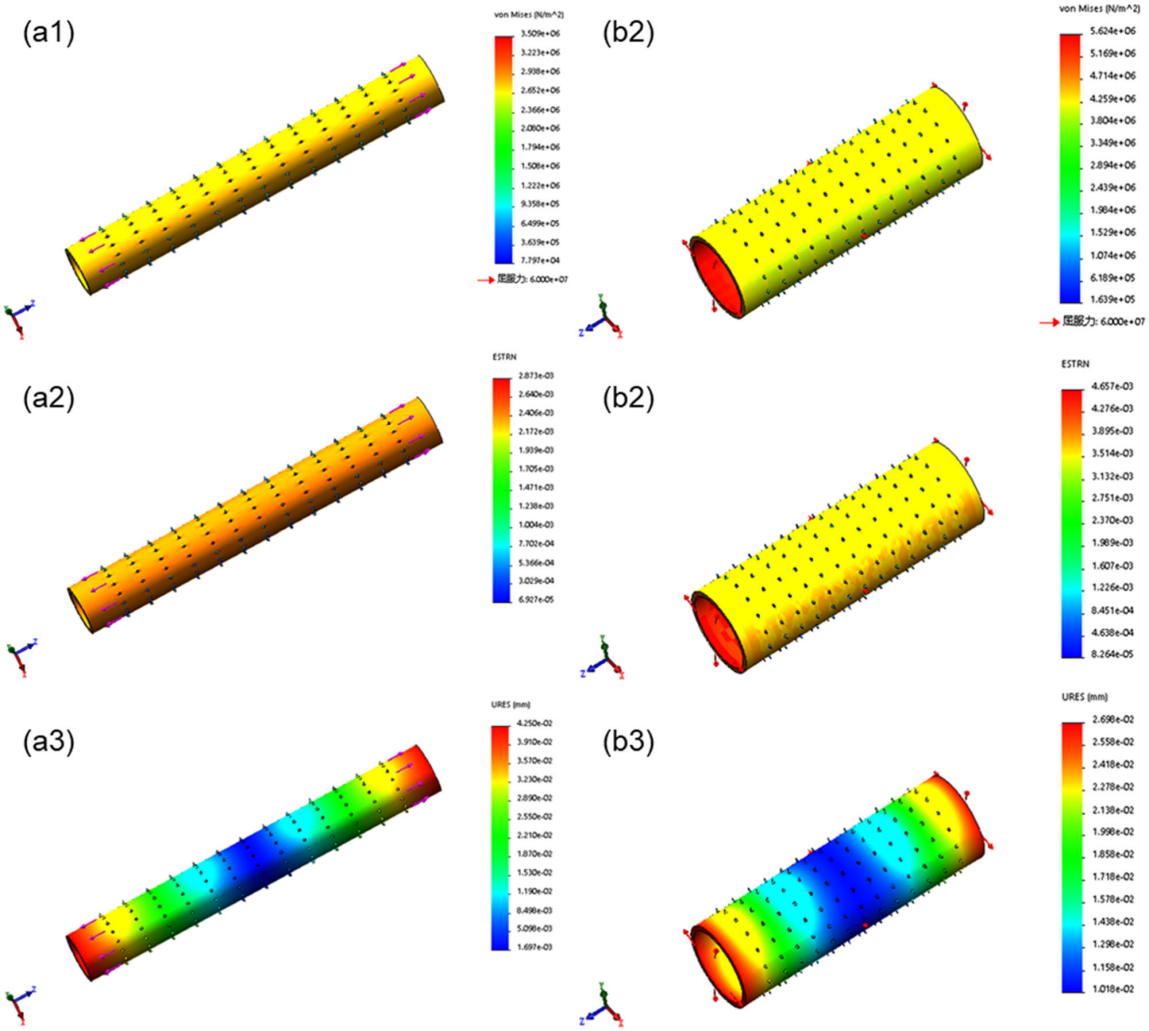
Mechanical simulation of bioartificial blood vessel with thorn-like protrusions: **a1** Axial stress. **a2** Axial strain. **a3** Axial displacement. **b1** Radial stress. **b2** Radial strain. **b3** Radial displacement

The stress and strain in the bioartificial blood vessel without thorn-like protrusions change uniformly, and there are no stress concentrations, whereas thorn-like protrusions produce stress concentrations (Figs. 5, 6). In the bioartificial blood vessel without thorn-like protrusions, the axial ultimate stress exceeds 6.8 MPa, the radial ultimate stress exceeds 0.9 MPa, the axial ultimate strain exceeds 4%, and the radial ultimate strain exceeds 1.6%. The radial limit displacement is greater than 0.139 mm and the radial limit displacement exceeds 0.039 mm. Spinous processes have a large impact on the axial ultimate stress of the bioartificial blood vessel, whereas the radial ultimate stress, strain, and displacement have a smaller impact.

From the above analysis, the thorn-like protrusions will produce greater stress concentrations and weaken the mechanical properties of the bioartificial blood vessel. Therefore, in subsequent tests, only the biomechanical properties of type B bioartificial blood vessels that were electrospun using 1 wt% Tween 80 in the electrospinning solutions were considered.

### Mechanical properties

**Fig. 7:**
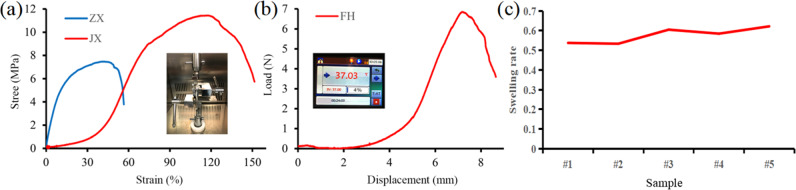
Mechanical properties of bioartificial blood vessel: **a** Tensile performance. ZX and JX are the axial tensile curve and radial tensile curve, **b** Load-displacement curve for suture force. FH is the suture force curve of the cross-linked bioartificial blood vessel, **c** Swelling rate for the different groups

The axial ultimate stress of the bioartificial blood vessel is 7.48 MPa and the radial ultimate stress is 11.44 MPa (Fig. 7a). Compared with the maximum stress experienced by the human abdominal aorta and coronary arteries [40, 41], the mechanical properties of the bioartificial blood vessel meet the requirements for clinical transplantation (Table 1).

**Table 1:** Tensile strength of bioartificial blood vessel

Vessel	Stress (MPa)	
	Axial ultimate stress	Radial limit stress
Abdominal aorta	1.47	5.29
Coronary artery	1.30	1.43
Bioartificial blood vessel	7.48	11.44

The maximum suture force of the bioartificial blood vessel is 6.852 ± 0.61 N (Fig. 7b), which is higher than the maximum value of 1.47 ± 0.49 N of the human mammary artery and the maximum value of 1.76 ± 0.45 N of the human great saphenous vein, thus meeting clinical needs in terms of suture force [42, 43].

According to Equation (1), the blasting strength of the bioartificial blood vessel is 915.2 kPa and about 6881.2 mmHg, which is higher than the human body’s ability to withstand internal pressure (1680 ± 307 mmHg, about 223 ± 40.8 kPa) [44, 45].

According to Equation (2), the swelling rate of the bioartificial blood vessel is 57.65% (Fig. 7c). Since PLCL is hydrophobic and collagen is hydrophilic, after cross-linking treatment, the shape of the bioartificial blood vessel is relatively stable without significant volume change.

It can be seen from the above analysis that the bioartificial blood vessel has excellent mechanical properties.

### Cell compatibility

**Fig. 8:**
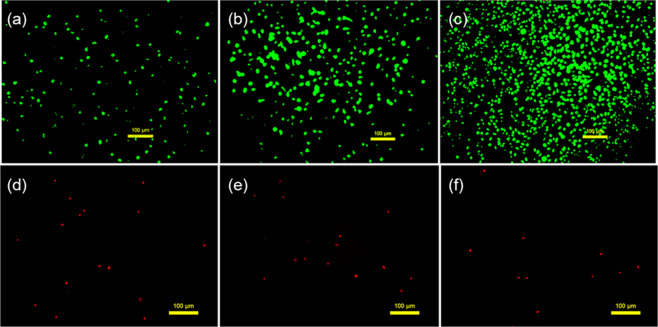
Live/Dead staining of HUVECs cultured on the bioartificial blood vessel: **a** Live cells on the bioartificial blood vessel at day 2. **b** Live cells on the bioartificial blood vessel at day 4. **c** Live cells on the bioartificial blood vessel at day 7. **d** Dead cells on the the bioartificial blood vessel t day 2, **e** Dead cells on the bioartificial blood vessel at day 4, **f** Dead cells on the bioartificial blood vessel at day 2, **e** Dead cells on the bioartificial blood vessel at day 7

**Fig. 9:**
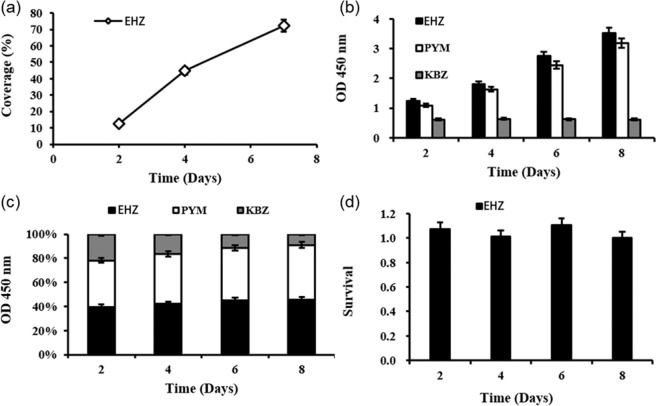
Statistical analysis of HUVEC growth on the bioartificial blood vessel: **a** Cell coverage of the bioartificial blood vessel, EHZ represents the cross-linked group. **b** OD values from CCK-8 test, PYM represents the petri dish group and KBZ represents the blank control group. **c** Stacked histogram of OD values. **d** Cell viability of HUVECs

Throughout the cell culture process, the number of live cells on the bioartificial blood vessel gradually increased. Cell coverage reached 75.09 ± 1.65% on day 7 and the number of dead cells was minimal (Figs. 8, 9a).

The CCK-8 cell proliferation and toxicity test results show a higher proliferation rate for HUVECs on the bioartificial blood vessels compared to the cells in the tissue culture dish (Fig. 9b). The total number of cells on the bioartificial blood vessel was higher than the total number of cells in the petri dish (Fig. 9c).

The cell survival rate indicates that cells proliferate faster during the first few days of culture, and the proliferation rate begins to slow on day 8. A possible reason for this is that the cell density is greater on day 8, which may lead to contact inhibition. The proliferation rate was higher than that of cells in the petri dish for up to 8 days (Fig. 9d), indicating that the bioartificial blood vessel may promote cell proliferation.

**Fig. 10:**
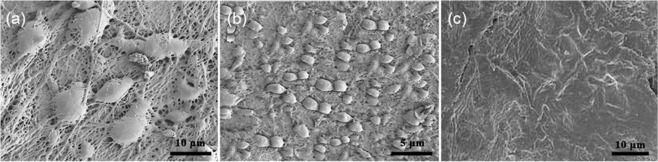
Representative scanning electron micrographs of HUVECs on bioartificial blood vessels: **a** Day 2. **b** Day 4. **c** Day 9

Representative SEM images of cells on the bioartificial blood vessel show that the cells have a spindle-shaped appearance on day 2, suggesting that the cells have begun to differentiate (Fig. 10a). After, the cells continue to multiply (Fig. 10b). At day 9, a monolayer of HUVECs can be observed (Fig. 10c).

From the above analysis, it can be concluded that the bioartificial blood vessel promotes cell adhesion and proliferation, indicating excellent cell compatibility.

### In vivo compatibility

1. Bioartificial blood vessel implantation
  During the implantation process (Fig. 11a–e), the bioartificial blood vessel has a better anastomosis than an expanded polytetrafluoroethylene (e-PTFE) bioartificial blood vessel and no blood leakage at the suture site. Blood flow through the sutured bioartificial blood vessel was detected using color Doppler ultrasound. The blood flow was smooth, and the flow rate was normal (Fig. 11f).

**Fig. 11:**
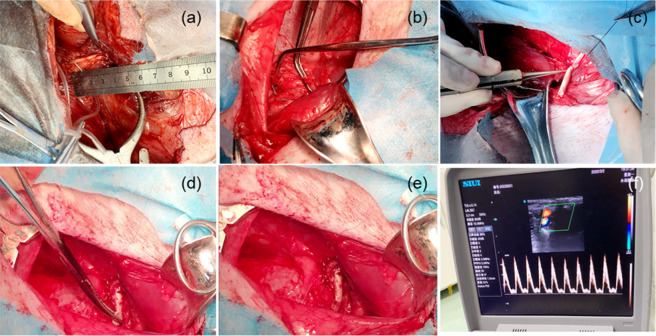
Implantation of a bioartificial blood vessel in a pig: **a** Exposure of the left posterior femoral artery. **b** Peeling and clamping of the femoral artery. **c** Surgical suturing of the bioartificial blood vessel. **d** Release of one end of the arterial clip, **e** Full release of the arterial clip and completion of the implantation process, **f** Color Doppler ultrasound to detect blood flow
  The results show that the bioartificial blood vessel has excellent mechanical properties during implantation.
2. Analysis of bioartificial blood vessel removal
  The bioartificial blood vessel was implanted in experimental pigs for four weeks. Before removing the bioartificial blood vessel (Fig. 12), the surgical suture site recovered well and no infection or swelling occurred. The color Doppler ultrasound showed no thrombosis or blockage in the bioartificial blood vessel at the implantation site and blood flow was normal (Fig. 12h, i). Cutting the bioartificial blood vessel along the middle, it was found that the outer wall of the bioartificial blood vessel was wrapped in muscle-like tissue, the overall color was uniform, and there were no signs of necrosis and no debris attached to the inner wall. Observations with the naked eye were similar to the color Doppler ultrasound observations.

**Fig. 12:**
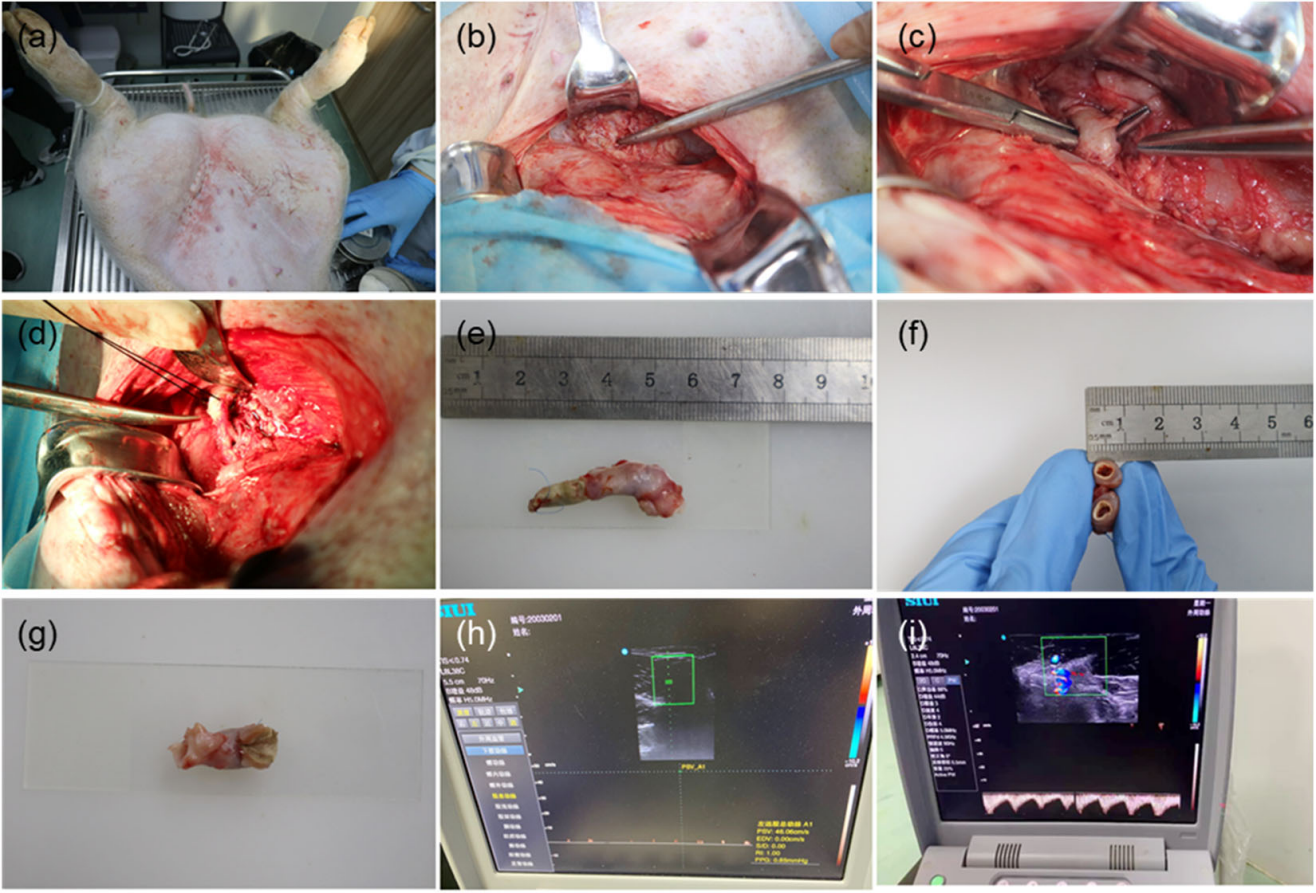
Removal of the bioartificial blood vessel: **a** Anesthetizing the pig to expose the surgical scar. **b** Peeling the tissue layer by layer. **c** Exposing the implanted bioartificial blood vessel. **d** Cutting the bioartificial blood vessel at both ends of the suture. **e** Removing the bioartificial blood vessel. **f** Cutting the bioartificial blood vessel along the middle. **g** Cutting the bioartificial blood vessel along the axial direction. **h** Color Doppler ultrasound image of the bioartificial blood vessel. **i** Blood flow through the bioartificial blood vessel
  The results show that the bioartificial blood vessel has excellent biocompatibility during implantation.
3. HE staining results
  The surface of the lumen of a host blood vessel consists of a smooth, continuous endothelium, with a small number of cells inside, which are tightly connected by extracellular matrix to form a continuous layer of endothelial cells (ECs). The cells of the blood vessel wall are clearly layered. (Fig. 13a1–a3).

**Fig. 13:**
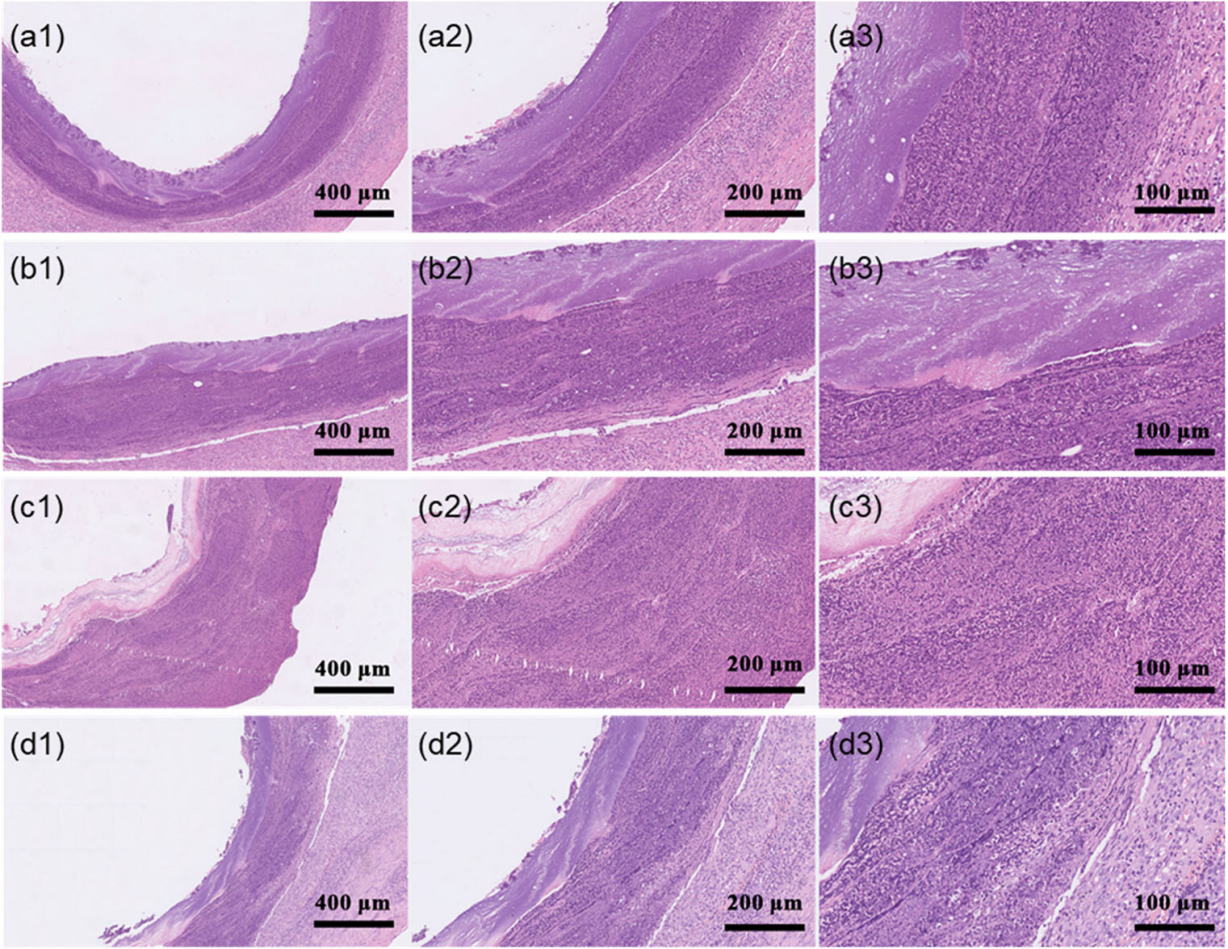
HE staining on bioartificial blood vessels (100x, 200x, and 400x magnification): **a1**–**a3** Pig blood vessel. **b1**–**b3** Proximal anastomotic bioartificial blood vessel. **c1**–**c3** Middle section of bioartificial blood vessel, **d1**–**d3** Distal anastomotic bioartificial blood vessel
  New tissue on the inner membrane can be observed on the surface of the lumen of the bioartificial blood vessel. The surface is covered by a single layer of cells, which are connected to each other by extracellular matrix, exhibiting an ordered nuclear structure. However, the monolayer is not as smooth and the cells are not as tightly connected or slender as those of the host blood vessel. Infiltration of cells into the bioartificial blood vessel is more obvious near the anastomotic position compared to the middle. A possible reason for this is that suturing at the anastomosis stimulates the host blood vessel, causing more cells to infiltrate the bioartificial blood vessel and proliferate [46, 47]. The cell nuclei were stained blue and can be clearly observed at 400x magnification (Fig. 13b1–d3).
  Overall, new tissue appeared on the surface of lumen of the bioartificial blood vessel after the vessel was implanted in the pig body. This new tissue formation occurred as a result of cells gradually infiltrating from the anastomosis at both ends and migrating towards the middle of the vessel. With continuous infiltration of cells and continuous degradation of the bioartificial blood vessel, the bioartificial blood vessel will gradually become a real blood vessel in the host.
4. VEGF staining results

**Fig. 14:**
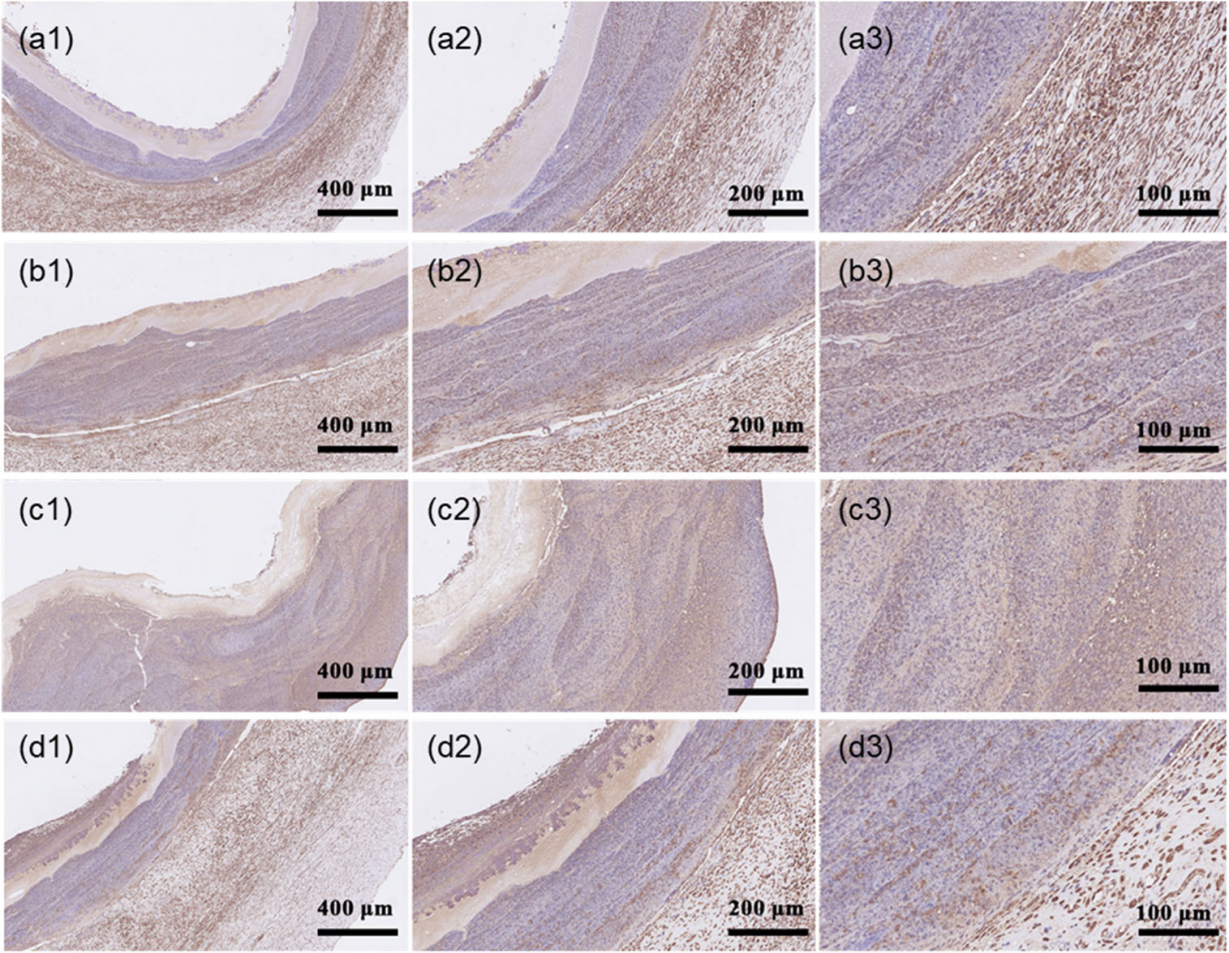
VEGF staining of bioartificial blood vessels (100x, 200x, and 400x magnification): **a1**–**a3** Host blood vessel. **b1**–**b3** Proximal bioartificial blood vessel. **c1**–**c3** Mid-section of bioartificial blood vessel, **d1**–**d3** Distal bioartificial blood vessel

The lumen of the pig blood vessel has a smooth continuous brown band and staining is evenly distributed (Fig. 14a1–a3). The lumen of the bioartificial blood vessel also has a continuous brown strip, although not as smooth as the pig blood vessel, indicating that many ECs have colonized on the inner surface. Excluding non-specific staining, coverage by ECs is orderly and dense.

The coloring depth of VEGF is higher at both ends of the bioartificial blood vessel than in the middle. The two ends are in contact with the suture therefore an inflammatory reaction occurs, which stimulates more secretion of VEGF by cells in the host blood vessel. The VEGF staining can be clearly seen at 400x magnification (Fig. 14b1–d3).

The results show that new tissue in the form of an EC layer covers the surface of the lumen of the implanted bioartificial blood vessel.

It can be seen from the above analysis that the bioartificial blood vessel smoothly circulates blood in pigs without thrombosis. HE staining, immunoenzymatic labeling for VEGF, and other results show that the bioartificial blood vessel has excellent in vivo compatibility.

## Conclusions

In this study, simulations of thorn-like protrusions on electrospun scaffolds were carried out. Thorn-like protrusions produce stress concentrations in the bioartificial blood vessel and weaken its mechanical performance. Nanofibers with a clear core/shell structure were prepared and the influence of 1 wt% Tween 80 in the electrospinning solution on the preparation of bioartificial blood vessels was analyzed. Bioartificial blood vessels without thorn-like protrusions and a clear core/shell structure were obtained. This solves typical problems with the electrospinning of bioartificial blood vessels. The effectiveness of Tween 80 was verified. Based on the macro and microscopic morphology, hydrophilicity, mechanical properties, cell compatibility, and in vivo test results, it can be concluded that the bioartificial blood vessels have excellent biomechanical properties. The results demonstrate the potential of bioartificial blood vessels prepared by electrospinning, which may be suitable for clinical applications.
